# Beta-arrestin 1 regulation of reward-motivated behaviors and glutamatergic function

**DOI:** 10.1371/journal.pone.0185796

**Published:** 2017-10-03

**Authors:** Nitish Mittal, Ani Minasyan, Nicole Romaneschi, Joshua K. Hakimian, Gabriel Gonzalez-Fernandez, Ralph Albert, Nina Desai, Ian A. Mendez, Timothy Schallert, Sean B. Ostlund, Wendy Walwyn

**Affiliations:** 1 Department of Psychiatry and Biobehavioral Sciences, Semel Institute, David Geffen School of Medicine, University of California Los Angeles, Los Angeles, CA, United States of America; 2 Division of Pharmacology and Toxicology, College of Pharmacy, The University of Texas at Austin, Austin, TX, United States of America; 3 Department of Anesthesiology and Perioperative Care, School of Medicine, University of California, Irvine, UCI Center for Addiction Neuroscience, School of Biological Sciences, University of California Irvine, Irvine, United States of America; 4 Brain Research Institute, University of California Los Angeles, Los Angeles, CA, United States of America; University of South Carolina School of Medicine, UNITED STATES

## Abstract

The two highly homologous non-visual arrestins, beta-arrestin 1 and 2, are ubiquitously expressed in the central nervous system, yet knowledge of their disparate roles is limited. While beta-arrestin 2 (βarr2) has been implicated in several aspects of reward-related learning and behavior, very little is known about the behavioral function of beta-arrestin 1 (βarr1). Using mice lacking βarr1, we focused on the role of this scaffolding and signal transduction protein in reward-motivated behaviors and in striatal glutamatergic function. We found that βarr1 KO mice were both slower in acquiring cocaine self-administration and in extinguishing this behavior. They also showed deficits in learning tasks supported by a natural food reward, suggesting a general alteration in reward processing. We then examined glutamatergic synaptic strength in WT and KO medium spiny neurons (MSNs) of the Nucleus Accumbens (NAc) shell in naïve animals, and from those that underwent cocaine self-administration. An increase in the AMPA/NMDA (A/N) ratio and a relative lack of GluN2B-enriched NMDARs was found in naïve KO vs WT MSNs. Applying Lim Domain Kinase (LIMK1), the kinase that phosphorylates and inactivates cofilin, to these cells, showed that both βarr1 and LIMK regulate the A/N ratio and GluN2B-NMDARs. Cocaine self-administration increased the A/N ratio and GluN2B-NMDARs in WT MSNs and, although the A/N ratio also increased in KO MSNs, this was accompanied by fewer GluN2B-NMDARs and an appearance of calcium-permeable AMPARs. Finally, to examine the consequences of reduced basal GluN2B-NMDARs in reward-processing seen in KO mice, we chronically infused ifenprodil, a GluN2B antagonist, into the NAc shell of WT mice. This intervention substantially reduced food-motivated behavior. Together these findings identify a previously unknown role of βarr1 in regulating specific reward-motivated behaviors and glutamatergic function.

## Introduction

The non-visual arrestins, beta-arrestin 1 and 2, also known as arrestin 2 and 3 respectively, are ubiquitously expressed scaffolding and signal transduction proteins involved in diverse cellular functions. Beta-arrestin 2 (βarr2) plays a central role in the rewarding profiles of morphine, cocaine and alcohol [1–4]. In contrast, very little is known of the role of Beta-arrestin 1 (βarr1) in reward-motivated learning and behavior [5].

Both of these isoforms regulate remodeling of the actin cytoskeleton to influence chemotaxis [6–9] and trafficking of specific G-protein coupled receptors [10–12]. The actin cytoskeleton also controls the trafficking and function of ionotropic glutamatergic receptors. The trafficking and stability of AMPARs [2-amino-3-(3-hydroxy-5-methyl-isoxazol-4-yl) propanoic acid receptors] is regulated by the actin depolymerizing factor/cofilin pathway [13–16]. GluN2B -containing NMDARs (N-methyl D-aspartate receptor subtype 2B) also rely on the actin cytoskeleton and associated microtubules for externalization [17, 18]. In providing a scaffold for cofilin, Lim domain kinase (LIMK) and slingshot (SSL), or chronophin (CIN) to regulate the actin cytoskeleton [9, 19, 20], βarr1 may also control the trafficking and thus function of these glutamatergic receptors.

Cocaine-induced alterations in glutamate signaling are particularly evident in the shell of the NAc [21, 22], a region that is also important for aspects of food-motivated behavior [23, 24]. While ionotropic AMPA-type glutamate receptors (AMPARs) dominate excitatory glutamatergic transmission in the NAc shell, NMDA-type glutamate receptors (NMDARs) also make important contributions to synaptic plasticity [25]. Drugs of abuse can lead to persistent alterations in the subunit composition of both of these receptors; prolonged withdrawal from chronic cocaine exposure upregulates GluR1 containing AMPARs in the NAc shell [26–28] and repeated cocaine exposure leads to a short-term increase in so-called silent synapses [29, 30], which are rich in GluN2B-containing NMDARs and relatively poor in AMPARs [25].

We propose that βarr1, in providing a scaffold for these proteins that regulate actin turnover, may influence basal glutamatergic signaling and cocaine-induced adaptations of this excitatory system within the NAc shell. This would alter select aspects of reward processing. We have used an array of behavioral and electrophysiological assays to examine this hypothesis and, in doing so, have defined specific roles of βarr1 and NMDARs in reward processing.

## Methods

### Animals

All experiments were conducted in accordance with the AALAC Guide for the Care and Use of Laboratory Animals and the Policies on the Use of Animals in Neuroscience Research established by the Society for Neuroscience. All experiments were approved by the Office of Animal Research Oversight (OARO; protocol numbers 2008–111 and 1999–179) at UCLA before proceeding. Mice lacking βarr1[31] were fully back-crossed into the C57Bl/6 background and mice lacking βarr2 on a C57Bl/6 background were obtained from Jackson laboratories (Bar Harbor, MA; stock# 011130). For all experiments, two to three months old, equally distributed, male and female mice from heterozygous matings were used. For all surgical procedures. isoflurane (2% for induction and 1% for maintenance) was used to induce and maintain anesthesia. After these surgeries, mice were placed in their pre-warmed home cage and received carprofen gels, a non-steroidal anti-inflammatory to treat pain and inflammation, and 3–4 nestlets to create their bed. At the end of each behavioral experiment or prior to electrophysiology recordings in naïve mice, all mice were deeply anesthetized by isoflurane (5%) and decapitated, some of these mice were then used for electrophysiology experiments or to determine cannulae placement. Mice that were removed from the study for any reason were similarly anesthetized by isoflurane (5%) and euthanized.

### Intravenous self-administration

An intravenous catheter (0.2 mm i.d., 0.4 mm o.d.; Cathcams, Oxford, UK) was inserted into the right jugular vein of mice under sterile conditions as previously described [32]. After heart rate and breathing increased following the surgery, the mouse was placed in a pre-heated cage on a heating pad (Sunbeam Products Inc, Boca Raton, FL, USA) until sternal and normal behavior observed. During the 7-day recovery period and for the duration of the experiment, the catheter was flushed with 0.02 ml heparin/saline (30 USP/ml), and Timentin (67 mg/ml). The mice were monitored twice daily for the first 48h after surgery and daily thereafter for the duration of the experiment for loss of body weight and/or a moribund state. Exclusion criteria of more than a 15% weight lost or a moribund state for more than24h was applied and the mice removed from the study if this criteria was met. After 7 days of recovery, the mice underwent daily self-administration of cocaine (1 mg/kg/infusion), or saline, in operant conditioning chambers in which the active and inactive levers were randomly assigned to the right and left levers (Med-Associates Georgia, VT). A response on the active, but not inactive, lever resulted in an intravenous drug infusion (0.67μl/g body weight) and presentation of a 20-s tone and visual light cue. A 20-s ‘timeout’ period, during which no drug could be earned, followed each drug infusion. To facilitate exploration of the levers, a droplet of 20% sweetened condensed milk was placed on both the active and inactive levers for all mice, three times per session during the first two sessions. The mice had *ad libitum* access to food during the IVSA procedure. The mice underwent a minimum of ten days of acquisition training at fixed ratio one (FR1) during which one lever press delivered one drug infusion, followed by five days at fixed ratio two (FR2) and 5 days at fixed ratio five (FR5), where two and five lever presses respectively resulted in a single infusion. Self-administration behavior was considered established at each schedule if a minimum of ten infusions were obtained per session and there was no more than 20% variation in the number of infusions earned over three consecutive days during FR1, FR2 and FR5. Catheter patency was tested using an infusion of propofol (20 μl of 1% w/v in saline) every five days. Preceding data from mice that did not respond to propofol were removed from the study. Mice that successfully completed acquisition through FR5 underwent extinction training under an FR1 schedule. This followed the same protocol as during FR1 except saline, not cocaine was delivered in response to a press on the active lever.

#### Statistical analysis

The data for the acquisition phase are represented as the number of infusions earned or active lever presses and were analyzed using a repeated measures ANOVA (Prizm v7). The data for the extinction phase are represented as the total number of lever presses and are also analyzed using a repeated measures ANOVA (Prizm v7). The loss of mice at each stage of this experiment and reasons for this loss is shown in S1 Table.

### Food-reinforced behavior

In this experiment, we applied a sophisticated behavioral approach that allowed us to characterize food-motivated Pavlovian and instrumental conditioning, as well as the ability for Pavlovian reward-predictive cues to selectively motivate instrumental reward-seeking actions based on a shared outcome (reward). This phenomenon, referred to as outcome-specific Pavlovian-to-instrumental transfer (PIT), has been extensively studied in both rats and mice [33–37], and is known to depend on NAc shell function [23, 38, 39]. Mice (WT: n = 9; KO: n = 7) were placed on a food deprivation schedule to maintain them at approximately 85% of their free-feeding body weight throughout training and testing. All behavioral procedures were conducted in a set of four identical behavioral chambers (Med Associates, Burlington, VT) equipped with two retractable levers located on either side of a recessed food port. Behavioral training began with 9 daily sessions of Pavlovian conditioning in which two auditory cues (85dB white noise and 2kHz pure tone) were paired with distinct food rewards (20-mg grain-based pellets, Bio-serv, New Jersey, or 0.02 ml of 20% sucrose solution). Half of the mice from each genotype received white noise-grain and tone-sucrose pairings, whereas the remaining mice received the opposite pairings. Cue presentations were 2 min in duration, during which the appropriate reward was delivered on a random time 30-sec schedule. Sessions consisted of 4 trials with each cue (8 total), with cue presentations separated by a variable 3-min interval. Trial order varied randomly over days. Pavlovian conditioned approach behavior was quantified as the number of food port entries performed per minute during conditioned stimulus (CS) presentations, excluding any component of the CS trial that followed a reward delivery to avoid the inclusion of entries related to unconditioned reward consumption. Food port entries during 2-min pre-CS periods served as a baseline measure. Following the Pavlovian conditioning phase, mice were given 11 days of instrumental training to perform two different lever-press actions (left and right) for distinct food rewards (grain and sucrose). For half of the mice in each genotype condition, the left press produced grain and the right press produced sucrose, whereas the remaining mice were trained with the opposite contingencies. Instrumental and Pavlovian contingencies were counterbalanced. Mice were trained with each lever-press action in separate daily sessions. Each session began with the insertion of the appropriate lever and ended once 15 rewards had been earned or until 30 min had elapsed. Sessions were separated by at least 2 hours. Lever pressing was continuously reinforced for the first 2 days of training. The reinforcement schedule was then shifted in 3-day increments through random ratio (RR)-5 (probability of reward = 0.2), RR-10 (probability of reward = 0.1), and RR-20 (probability of reward = 0.05). Mice then underwent PIT testing to assess the ability of the food-paired cues to influence instrumental reward-seeking behavior. During transfer test sessions, mice were given continuous access to both levers. Lever-press responses were recorded but were not reinforced. Food port entries were also continuously recorded. Each session consisted of 4 non-contingent presentations of each of the two auditory cues (2-min each) using a pseudo-random test order (ABBABAAB). The first cue was presented 8 min into the session, and cue presentations were separated by a fixed 4-min interval. Mice underwent two identical transfer test sessions, with 1 day each of Pavlovian and instrumental retraining between these sessions, conducted in that order. Retraining sessions were identical to those used during initial training, except that schedule used for instrumental retraining was rapidly shifted from continuous reinforcement, to RR-5, to RR-10 (1 reinforced lever press with each schedule), before reaching RR-20, which was used for the remainder of the session.

#### Statistical analysis

Data from this experiment were analyzed using mixed ANOVAs with genotype as a between-subject factor and appropriate within-subject factors (Prizm v7). For Pavlovian conditioning, we assessed the rate of food magazine entries (responses per minute) during pre-CS (2-min baseline) and CS periods (from CS onset to first reward delivery) over training days (1–9). Instrumental training data were analyzed as the mean rate of lever pressing (responses per minute, averaged across sessions) over training days (1–11). To focus on group differences in the ability to adapt to more effortful (i.e., higher ratio) schedules of reinforcement, a separate ANOVA was conducted on the mean rate of pressing for the first, second, and third session with each new RR schedule, averaging across RR5, RR10, and RR20 training sessions. During the transfer test, we assessed the rate of lever pressing during Pre-CS and CS periods, separating CS response rates according to whether a response was paired with the same reward as the CS or a different reward (Pre, Same, and Different). The mean rate of magazine entries during Pre-CS and CS periods (collapsed across CSs) during the transfer test was also assessed. Bonferroni correction was used for post-hoc tests.

### Electrophysiology

#### Slice preparation

Naïve mice, or self-administering mice 24h following the last session, were anesthetized by isoflurane (5%)and decapitated. Brains were rapidly extracted and 300 μm coronal slices cut on a vibratome (VT1000S, Leica Microsystems, Wetzlar, Germany) in an oxygenated, ice cold, potassium-gluconate solution containing (in mM): 140 K-gluconate, 15 Na^+^ gluconate, 4 NaCl, 10 HEPES, 0.2 EGTA, pH 7.2, 209–310 mOsm. The slices were then incubated in artificial cerebrospinal fluid (ACSF); in mM: 130 NaCl, 26 NaHCO_3_, 3 KCl, 2 MgCl_2_, 1.25 NaHPO_4_, 2 CaCl_2_, and 10 glucose pH: 7.4, osmolality: 300–310 mOsm., and continuously perfused with 95% O_2_-5% CO_2_ at room temperature (22–25°C) for one hour prior to recording.

#### Slice recordings

A Slicescope, (Scientifica, UK), consisting of an upright, modified Olympus BX51W1 microscope, manipulators and controllers coupled with an Axopatch 200B amplifier, NidAQ digitizer and winEDR (University of Stratchlyde, Glasgow, Scotland) software were used to obtain recordings. Borosilicate glass capillaries (World Precision Instruments, Sarasota, FL) were pulled using a micropipette puller (P-97, Sutter Instruments Company, Novato, CA) to a resistance of 3–6 MΩ, as measured by the recording electrode, when filled with intracellular solution (in mM): 125 Cs-methanesulfonate, 3 KCl, 4 NaCl, 1 MgCl_2_, 5 Mg ATP, 9 EGTA, 8 HEPES, 1 GTP Tris, 10 phosphocreatine disodium and 0.1 leupeptin, pH 7.25–7.3, osmolality, 280–290 mOsm. Evoked EPSCs (eEPSCs) were recorded form medium spiny neurons in the NAc shell in the presence of the GABA_A_ receptor antagonist, bicuculline (BIC, 10 μM) in the external solution, while holding the membrane potential at -70 mV or +40 mV, to obtain AMPAR and NMDAR currents respectively. The slices were perfused with 1–2 ml/min, oxygenated, ACSF at room temperature. The AMPAR and NMDAR antagonists, 6-cyano-7-nitroquinoxaline-2, 3-dione (CNQX, 10 μM) and amino-5-phosphonovaleric acid, (AP-V, 50 μM) respectively were then added into the external solution to block their respective currents as needed. 1-Naphthyl acetyl spermine trihydrochloride (NASPM, 100μM, Sigma-Aldrich) or Ro25-6981 (μM, Tocris) were used to block calcium permeable (CP)-AMPARs or GluN2B-NMDARs respectively. MSNs within the NAc shell were visualized by infrared microscopy coupled with differential interference contrast microscopy and identified by their size (8–12μm), positive reversal potential and basic membrane properties. A concentric bipolar electrode (FH, Bowdoinham, ME) was placed at the same plane 50–100μM from the recorded cell. The slices were stimulated with a ~400 μA current at a frequency of 0.3 Hz and adjusted so as to obtain a submaximal current of~300pA at -70mV. All antagonists were perfused for 300s at -70mV before the stimulation protocol of 60s duration. The recordings were analyzed using WinEDR and WinWCP software (University of Stratchlyde, Glasgow, Scotland) and recordings in which the series resistance was more than 30 mΩ initially or drifted by more than 20% over time were excluded.

#### Statistical analysis

Basal membrane properties and stimulating currents was not different across genotype: membrane resistance; KO: 217 ± 26, WT: 226 ± 50mΩ, capacitance; KO: 73 ± 29, WT: 64 ± 8 pF, series resistance; KO: 19.4 ± 1.2, WT: 18.5 ± 1.1 mΩ, holding current; KO: -63 ± 20, WT: -40 ± 14pA, access conductance; KO: 24 ± 2, WT: 21 ± 2 ns, stimulating current; KO: 484 ± 27, WT: 462 ± 39μM. Data are presented as a ratio, or as a percentage of the peak current, and were analyzed using Student’s t-test for 2 datasets and mixed ANOVA’s for multiple data sets followed by either the Tukey’s, two-stage Benjamin, Krieger and Yekutieli procedure test, or Student’s t-test post-hoc tests (Prizm v7).

### Chronic bilateral intra-accumbal infusions and food-reinforced instrumental conditioning

Food deprived mice (n = 13) underwent the surgery to implant bilateral cannulae (28GA, 1mm separation distance, 4.75mm length, PlasticsOne, Roanake, VA) in the medial NAc shell. These were connected to vinyl tubing (0.69x1.14mm, PlasticOne), a 21g Y-splitter (PlasticsOne), and a single, pre-filled, osmotic minipump (Alzet Model 1002, Durect Corp, Cupertino, CA, reservoir volume = 100μl, flowrate = 0.19μl/h, ~14d delivery time). The pumps contained either the GluN2B receptor antagonist ifenprodil tartrate (0.183mg/ml, 0.228mM, SigmaAldrich, St. Louis, MO) or vehicle, 0.1% tartaric acid (0.0285mM, SigmaAldrich). As the pumps were connected to bilateral cannulae, each NAc shell would be infused at a flow-rate of ~96nl/h to deliver 18ng/h and 6.05μg of ifenprodil tartrate over ~14 days. This dose was based on private communication with the authors of Gore and colleagues [40]. Under sterile surgical conditions, the pump, with attached Y-splitter and vinyl tubing, was first inserted into a subcutaneous pocket over the nape of the neck. The mice were then placed in the stereotaxic frame and the bilateral cannula lowered into the NAc shell (A/P: +1.1mm, M/L: +/-0.05mm, D/V: 4.5mm) using standard stereotaxic methods. The cannulae were glued in place (Loctite 454), allowed to dry, connected to the pump and dental cement applied (Bosworth, Skokie, IL). Due to the limited time of drug infusion, a shortened behavioral protocol focusing on the acquisition and performance of instrumental reward-seeking behavior was used. Two days after surgery, mice began seven days of magazine training to familiarize them with the behavioral context and the 20% sucrose solution reward. Twenty μl of sucrose was delivered randomly during the 30-sec schedule for each of 8 x2 min sessions. Daily sessions were ~40-min in length. They were then given instrumental conditioning using a similar procedure as described above, except they were trained to perform a single lever-press action for 20μl of a 20% sucrose reward (one 30-min session per day). Lever pressing was reinforced on days 1 and 2 under an FR1 schedule, on days 3 and 4 under an RR5 schedule, and on days 5 and 6 under an RR10 schedule. After behavioral testing, the mice were euthanized with isoflurane and their brains extracted and sliced to verify cannula placement.

#### Statistical analysis

The data for instrumental training are represented as the rate of lever presses per minute and were analyzed using mixed ANOVAs (Prizm v7). The data for magazine entries are represented as the number of entries per minute and analyzed using mixed ANOVAs (Prizm v7).

## Results

### Deleting βarr1 alters specific aspects of the intravenous cocaine self-administration profile

**Fig 1:**
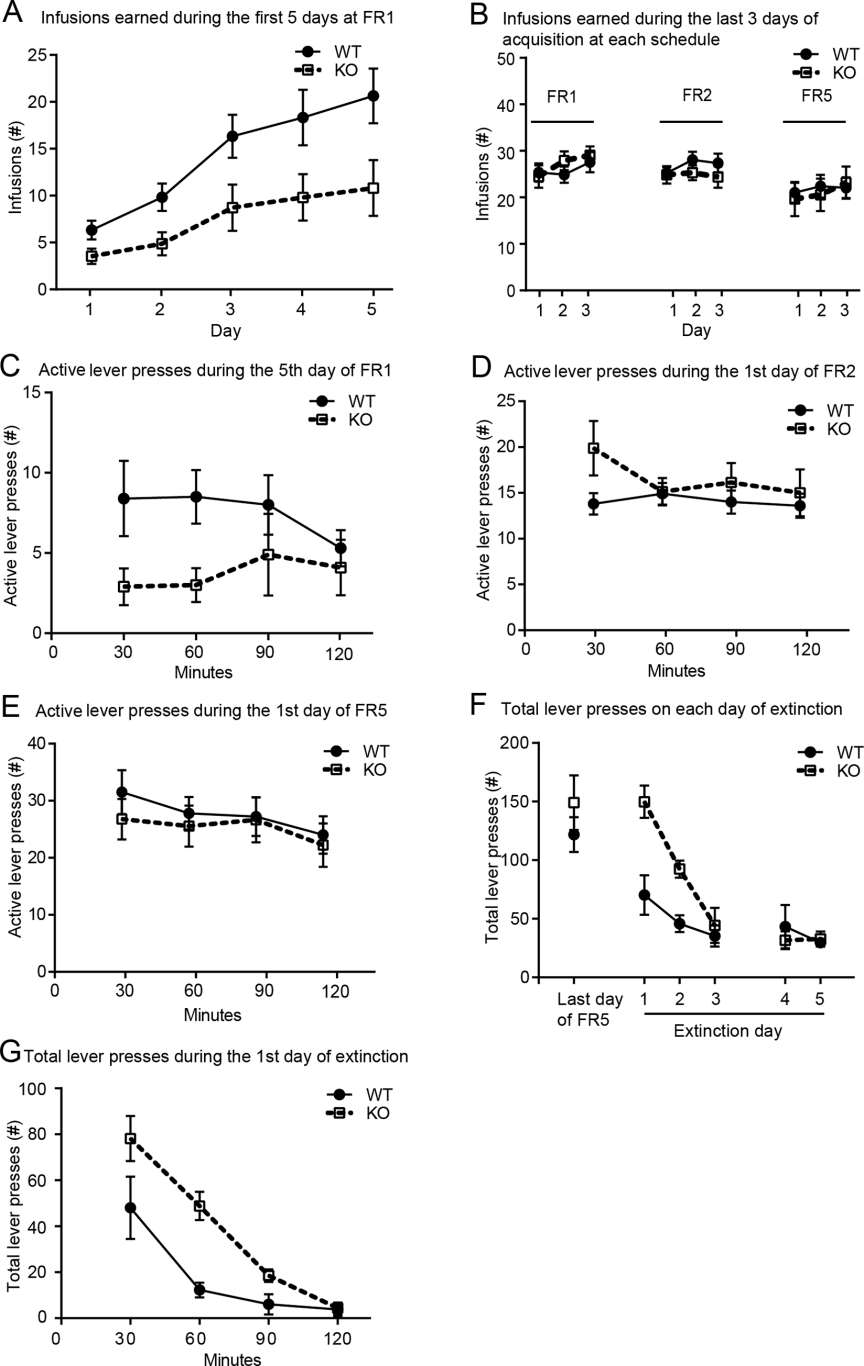
βarr1 KO mice show deficits in the acquisition and extinction of cocaine self-administration. **A)** The number of cocaine infusions earned by βarr1 KO mice (KO: n = 21) during the first 5 days of FR1 was lower than WT mice (WT: n = 22; p < 0.01). **B)** This difference in infusions earned was no longer present by the last three days of FR1 (WT: n = 22, KO: n = 19), FR2 (WT: n = 14, KO: n = 13) or FR5 (WT: n = 13, KO n = 11). **C, D** and **E.** Analysis of the number of within-session active lever presses during the fifth day of FR1 (**C**), the first day of FR2 (**C**) and FR5 (**D**) showed no effect of genotype. **F)** However, βarr1 KO mice (WT: n = 4, KO: n = 6) showed more persistent total lever pressing during the early phase of extinction (days 1–3; p < 0.01), but not thereafter (days 4–5). **G)** This effect was also seen during the first day of extinction when KO mice showed more persistent lever pressing during the first 90 min of this 120-min session.

In this experiment we first assessed the acquisition of cocaine self-administration behaviors under an FR1 schedule, and then progressed to FR2 and FR5 schedules to assess the motivation to obtain the drug. This was followed by 5 days of extinction, during which cocaine reinforcement was omitted but all response-contingent cocaine cues were presented under an FR1 schedule. The transition between each phase was based on specific criteria of stable lever pressing behavior (see methods). Using a repeated measures (RM)-ANOVA we saw a main effect of genotype as KO mice acquired cocaine self-administration behavior slower than WTs. This was evident during the first 5 days of FR1, where we saw a main effect of genotype (WT vs. KO: p < 0.01, F_1,41_ = 8.318; Fig 1A), but not during the last three days at this schedule (WT: n = 22, vs. KO: n = 19: p = 0.917, F_1,39_ = 0.001), nor during FR2 (WT: n = 14, vs. KO: n = 13: p = 0.396, F_1,25_ = 0.746), or FR5 (WT: n = 13, vs. KO: n = 11: p = 0.861, F_1,22_ = 0.0.031; Fig 1B) where no such effect of genotype was observed. Furthermore, survival analysis showed that WT mice reached criteria, as described in the methods, faster than the KO mice at FR1 (Gehan-Breslow-Wilkcoxon test: p < 0.05, χ^2^ = 6.479; S1 Fig). although there was no difference in the total number of days at FR1 (WT: 11.32 ± 1.04, KO: 14.05 ± 1.22), FR2 (WT: 5.43 ± 0.40, KO: 4.77 ± 0.32), or FR5 (WT: 6.46 ± 0.53, KO: 7.82 ± 0.87). Within-session analysis of specific days of FR1, FR2 and FR5 was examined by RM-ANOVA using 30-min time bins for the duration of the 2-hour sessions. This showed no main effect of genotype on the total number of active lever presses during the fifth day of FR1 (F_1,18_ = 1.655, p = 0.2145), the first day of FR2 (F_1,16_ = 1.222, p = 0.2854; Fig 1C) and FR5 (F_1,17_ = 0.324, p = 0.5768; Fig 1D) There was also no main effect of genotype on the association of the active lever with drug reinforcement, as defined by the % active lever presses (%(AL/(AL+IAL))), either during the first 5 days of acquisition at FR1 (WT vs. KO: p = 0.174, F_1,41_ = 1.914), the last three days of FR1 (F_1,18_ = 0.8093, p = 0.3802), FR2 (F_1,24_ = 1.507, p = 0.2315), or FR5(F_1,13_ = 0.9042, p = 0.3590).

The number of mice that were excluded, mostly due to a loss of catheter patency over time, are shown in S1 Table. As this could influence our results, we also analyzed these data using data from the mice with patent catheters at the end of FR5 (13 WTs and 11 KOs). Similar to the results from the complete dataset, we found a significant main effect of genotype (p < 0.05, F_1,22_ = 5.264) during the first 5 days of FR1. but no effect of genotype was observed for the last three days of FR1 (p = 0.757, F_1,22_ = 0.098) or FR2 (p = 0.458, F_1,22_ = 0.570) while the FR5 statistics remained unchanged as all subjects were included by this stage. This shows that the reported results were not influenced by the removal of subjects from either genotype over time.

Following stable acquisition at FR5, both groups decreased their lever-pressing behavior during the 5 days of extinction (p < 0.0001, F_4,32_ = 17.60; Fig 1E) and no effect of genotype was seen by the last 2 of the 5 days of this protocol (WT vs. KO: p = 0.723, F_1,8_ = 0.135). However, extinction was delayed in KOs who showed more persistent lever pressing behavior during the first 3 days of extinction and a main effect of genotype was observed (WT: n = 4 vs. KO: n = 6: p < 0.01, F_1,8_ = 15.64). Analysis of the within-session data for the first day of extinction reflected this delay with KOs showing a slower rate of extinction than WTs (p < 0.01, F_1,8_ = 13.31, Fig 1F).

In summary these results show that deleting βarr1 impairs the early phase of cocaine self-administration when this behavior is first being established, but does not prevent mice from acquiring stable asymptotic responding and motivation to obtain cocaine. KO mice show a similar impairment during the initial days of extinction, when they must learn to inhibit their cocaine-seeking behavior in response to a change in the action-drug contingency. These findings suggest that, although βarr1 may not be important for processing the rewarding, or reinforcing, properties of cocaine, this isoform is clearly involved in regulating the acquisition and extinction of instrumental cocaine self-administration.

### Deleting βarr1 disrupts food-motivated behavior but leaves the motivational influence of food-paired cues intact

**Fig 2:**
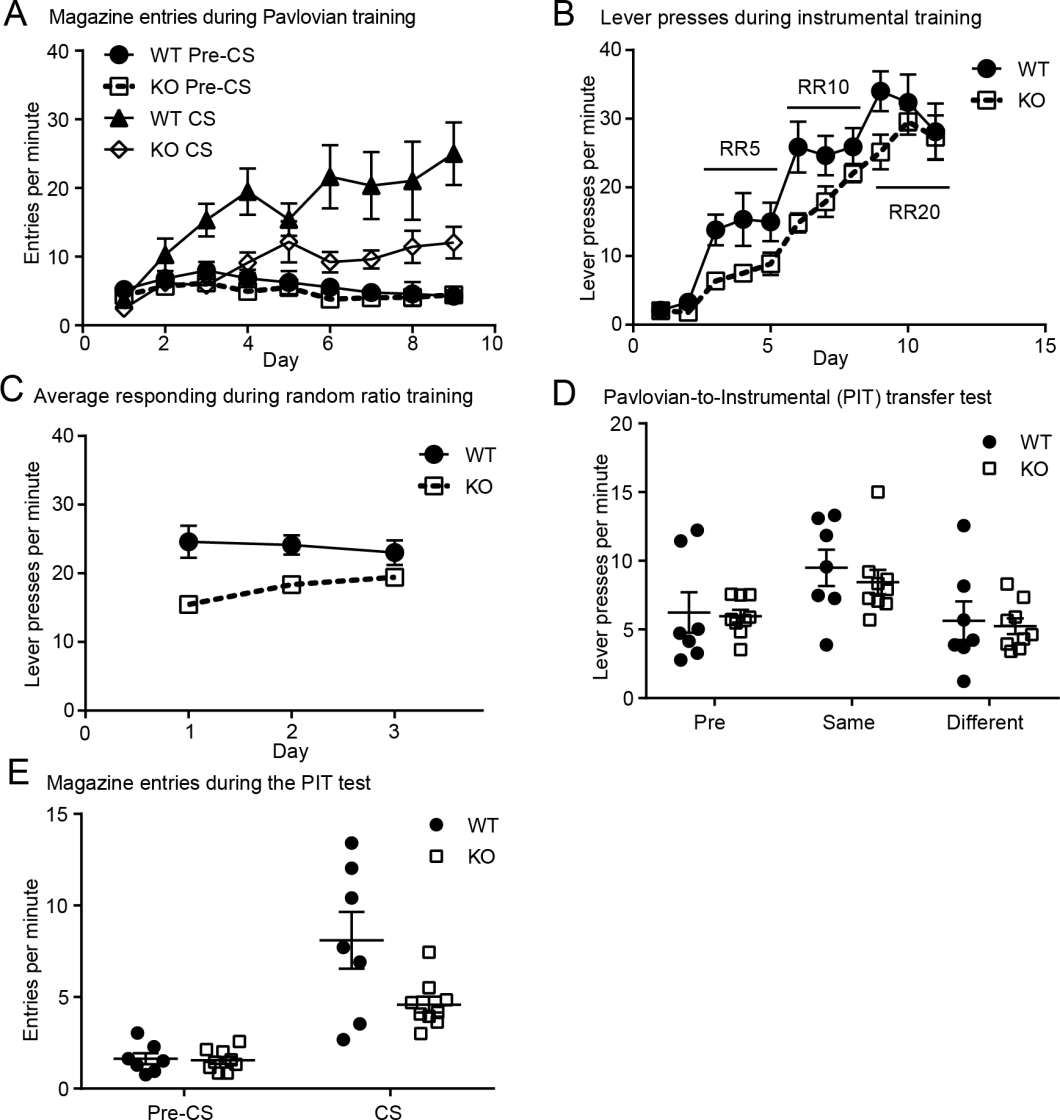
Deleting βarr1 disrupts food-reinforced Pavlovian and instrumental conditioning. **A**) Pavlovian conditioning was assessed over training days 1–9 by comparing the mean rate of food magazine entries performed during CS and Pre-CS (baseline) periods (WT: n = 7, KO: n = 9). KO mice showed a significantly lower asymptote of conditioned entry rate during CS periods. **B-C)** During instrumental conditioning, mice were allowed to earn food reward on progressively more effortful schedules of reinforcement (RR-5: Days 3–5; RR-10: Days 6–8; RR-20: Days 9–11). KO mice show lower rates of responding when adapting to more effortful schedules, and effect that was apparent in **B)** the mean rate of lever pressing over training days (1–11) and in **C)** the mean rate of responding on the first, second and third day of testing with each new level of RR schedule (collapsing across 3-day cycles with RR5, RR10, and RR20). **D)** Mean rate of lever pressing during the Pavlovian-to-instrumental transfer (PIT) test. During non-contingent CS presentations, both groups showed an appropriate increase in performance of Action Same, relative to their performance of Action Different and to their average rate of responding during the baseline (Pre-CS) period. **E)** Mean rate of magazine entries during the PIT test, plotted during the CS and Pre-CS periods. As during Pavlovian conditioning, KO mice showed attenuated conditioned magazine entry behavior during the CS, but not the pre-CS period as compared to the WT mice during PIT testing. *p<0.05 KO vs. WT.

To assess the scope of the behavioral impairments produced by the βarr1 deletion and to determine whether these impairments could be generalized to other rewarding stimuli, we conducted a series of experiments to characterize the performance of βarr1 KO mice in a battery of food-motivated behaviors. Mice were first given Pavlovian conditioning with two different stimulus-outcome contingencies. As can be seen in Fig 2A, all mice acquired conditioned approach behavior during Pavlovian conditioning–entering the food magazine at a higher rate during CS presentations than during pre-CS (baseline) periods. Importantly, although both groups acquired this conditioned response, KOs (n = 9) appeared to exhibit a lower behavioral asymptote than WTs (n = 7). Consistent with this interpretation, a Day (1–9) x Period (pre-CS vs. CS) x Group mixed ANOVA found significant effects of Day (Day F_8,112_ = 6.32, p < 0.001), Period (Period F_1,14_ = 45.41, p < 0.001), and Group (F_1,14_ = 7.51, p = 0.02), as well as significant Day by Period (F_8,112_ = 17.08, p < 0.0001), Period by Group (F_1,14_ = 10.58, p = 0.006), and Day by Period by Group (F_8,112_ = 2.88, p = 0.006) interactions. Further analysis (Day x Group ANOVA for each level of Period) revealed that the three-way interaction was driven by group differences in entry rates during CS periods (Day x Group: F_8,112_ = 2.15, p < 0.05; Group: F_1,14_ = 9.28, p < 0.01) but not during pre-CS periods (Day x Group: F_8,112_ = 0.46, p = 0.89; Group: F_1,14_ = 1.22, p = 0.29). Nevertheless, a Day x Period ANOVA performed on data from the KO group indicated that these mice also developed a significant elevation in food magazine entries during CS periods (Day: F_8,64_ = 2.79, p = 0.01; Period: F_*1*,*8*_ = 21.58, p = 0.002; Day x Period: F_8,64_ = 7.18, p < 0.001).

The next phase of the experiment, when mice learned to perform lever-press actions for action-specific food rewards (i.e. grain pellet or sucrose paired with left or right lever), is shown in Fig 2B. Here too, although both groups learned to respond for food, behavior was attenuated in KO mice. A Day (1–11) x Group mixed ANOVA performed on these data revealed significant effects of Day (F_10,140_ = 45.57, p < 0.001) and Group (F_1,14_ = 12.14, p = 0.004). Although the Day by Group interaction did not reach significance (F_10,140_ = 1.37, p = 0.20), it appeared that the group difference in press rates was more pronounced when the mice were first transitioned to more challenging random ratio schedules (Days 3, 6, and 9), forcing them to exert more effort to earn reward, relative to when they were more accustomed to those schedules (Days 5, 8, 11). To assess this effect more directly, we conducted a Day (1–3) x Group ANOVA on the average rate of responding during the first, second, and third day of random ratio training, averaging the data across each 3-day cycle (RR-5, RR-10, and RR-20; see Fig 2C). The analysis found no effect of Day (F_2,28_ = 1.15, p = 0.33) but did detect a significant Group effect (F_1,14_ = 12.34, p <0.01) and, more importantly, a significant Day x Group interaction (F_2,28_ = 4.61, p < 0.05). Bonferroni-corrected post-hoc testing indicated that the groups significantly differed during the first and second (p’s < 0.01), but not the third (p > 0.3), day of training with a new, more demanding RR schedule.

Mice were then administered a PIT test to assess the excitatory, outcome-specific influence of Pavlovian learning on their instrumental reward-seeking behavior. This influence can be seen in Fig 2D, which shows the mean rate of lever pressing during pre-CS and CS periods, separately plotting CS data for each lever-press action based on whether that action was trained with the same or a different reward as the CS. We found that both groups showed a selective elevation in performance of an action the CS that signaled the same reward as that action was non-contingently presented (Period Same), relative to trials with the alternate CS, which signaled a different food reward (Period Different), and baseline period (Pre-CS). A Period (Pre, Same, and Different) x Group mixed ANOVA found a significant effect of Period (F_2,28_ = 12.15, p < 0.001), but found no evidence of a main effect of Group or Period x Group interaction (F’s < 1, p’s > 0.7). Interestingly, as during initial Pavlovian conditioning, KO mice showed attenuated conditioned magazine entry behavior during the PIT test (Fig 2E). A Period (Pre vs. CS) x Group ANOVA found a significant effect of Period (F_1,14_ = 45.69, p < 0.001) and Group (F_1,14_ = 5.39, p < 0.05), as well as a significant Period by Group interaction (F_1,14_ = 5.91, p < 0.05). Although both groups exhibited a significant increase in magazine entries during the CS, relative to the pre-CS period (wildtype: t_*8*_ = 7.86, p < 0.001; knockout: t_*6*_ = 4.23, p < 0.01), their levels of responding significantly differed during the CS period (t_14_ = 2.44, p < 0.05) but not the pre-CS period (t_14_ = 0.27, p = 0.27).

In summary, KO mice showed attenuated goal-approach behavior to a food-paired cue and were also impaired in performing an effortful food-seeking task, particularly during initial transition periods with more challenging reinforcement schedules. While these findings implicate βarr1 in adaptive reward-motivated learning and behavior, the fact that KO mice showed relatively normal PIT performance suggests that βarr1 is not critical for assigning motivational or response-biasing properties to food-associated cues, thus demonstrating that these behavioral impairments were not the result of an underlying deficit in Pavlovian incentive learning.

### βarr1 regulates glutamatergic function in the NAc shell

So as to explore the molecular underpinnings of the behavioral impairments in reward-motivated behavior seen in KO mice, we performed a series of electrophysiological experiments. We focused on the glutamatergic system, a critical component of reward-related adaptations [41], within the NAc shell, a hub of reward-related signaling and glutamatergic function [26, 42].

#### The AMPA/NMDA ratio

**Fig 3:**
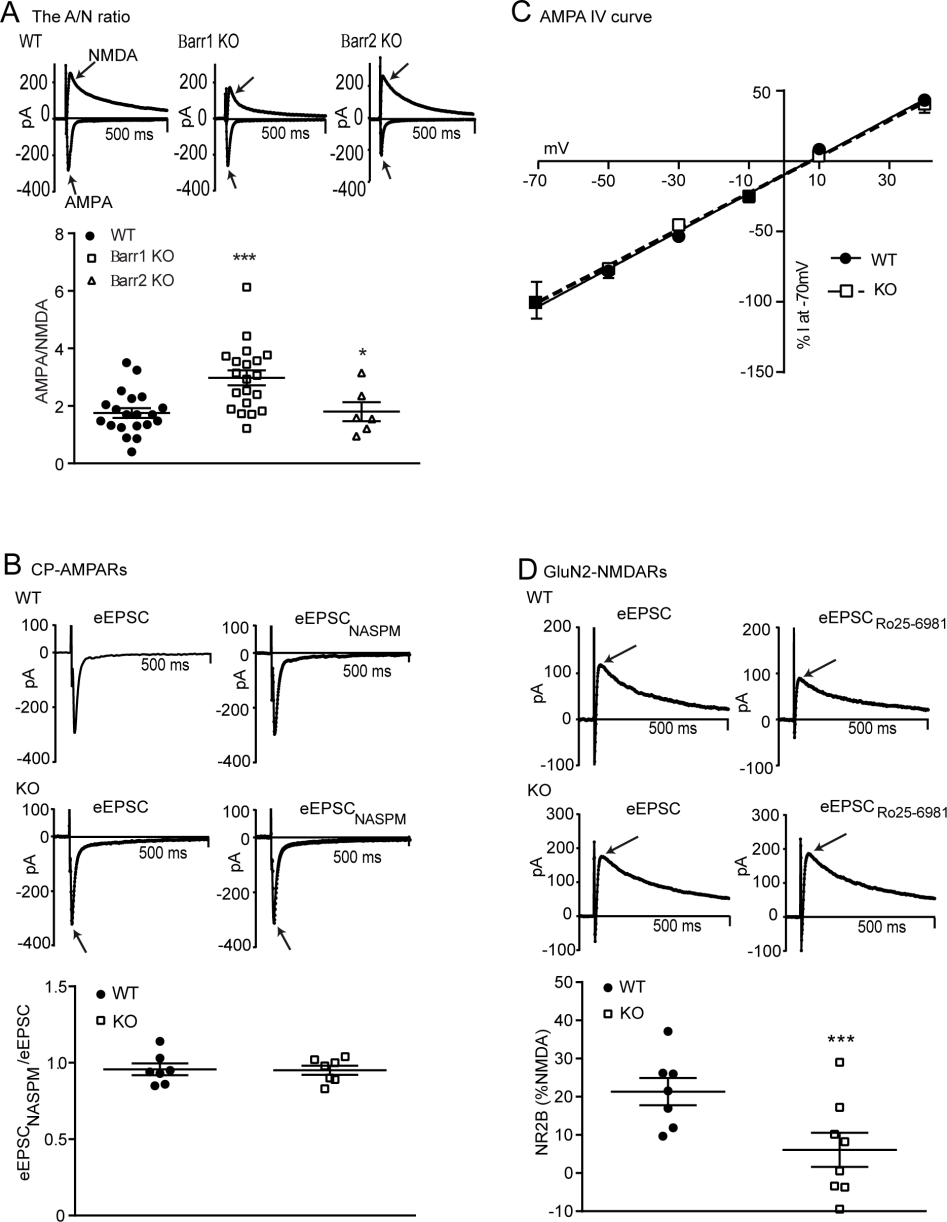
Deleting βarr1 alters glutamatergic synaptic strength in MSNs of the NAc shell. **A)** The A/N ratio was assessed in MSNs of the NAc shell by measuring: 1), the peak evoked AMPAR current at -70mV and, 2), the evoked NMDAR current, at +40mV and 45–55 ms following the peak eEPSC. The ratio of these currents was higher in βarr1 KO than WT MSNs and MSNs from βarr2 KO mice (WT: n = 20, βarr1 KO: n = 20, βarr2 KO: n = 6; *, ***p <0.05 and 0.001 respectively). **B)** CP-AMPAR currents, measured by the peak AMPAR current in the absence and then presence of NASPM at -70MV, showed no effect of genotype (WT: n = 4, KO: n = 7). **C)** The AMPA IV curve, assessed by the peak AMPAR current at -70, -50, -30, -10, 10 and 40 mV, and shown as a percent of the current at -70mV, also showed no effect of genotype (WT: n = 6, KO: n = 8). **D)** GluN2B containing NMDAR currents, measured as the peak NMDAR current at +40mV in the absence and then presence of Ro25-6981 (1μM), was reduced in KO, compared with WT, MSNs (WT: n = 7, KO: n = 8; p < 0.0001), *** = p < 0. 0001. Note that, in this and subsequent electrophysiology figures, the total length of the x-axis is 500ms and that stimulus artifacts were not removed from example traces.

A basal disruption of glutamatergic signaling, in particular that of AMPARs, may influence cocaine-seeking behaviors [43]. We therefore assessed basal glutamatergic synaptic strength, assessed by the A/N ratio, in WT, βarr1 and βarr2 KO MSNs. AMPAR currents were defined by the peak eEPSCs at -70mV in the presence of BIC whereas NMDAR currents were defined by the average current 45–55 ms after the peak eEPSC at +40mV in the presence of BIC. One-way ANOVA revealed a significant effect of genotype on the A/N ratio (WT: n = 20 cells, 6 mice, βarr1 KO: n = 20 cells, 5 mice, βarr2 KO: n = 6 cells, 2 mice; p < 0.0001) and post-hoc analysis showed that MSNs a higher A/N ratio in βarr1 KO mice compared with that of WT and βarr2 KO mice (Fig 3A).

#### AMPAR subunit composition

The increase in the A/N ratio could be a result of altered subunit composition of either AMPA or NMDA receptors [44]. For AMPARs this may be a result of altered trafficking and therefore function of GluR2-lacking CP-AMPARs [16]. As these receptors, containing GluR1, can be inhibited by NASPM, we assessed the peak evoked AMPAR current at -70 mV, in the presence of BIC and AP-V without, and then with, NASPM. We found that NASPM did not alter the peak AMPAR current in WT (n = 7 cells, 3 mice) or KO (n = 7 cells, 4 mice) MSNs (WT vs. KO: p = 0.804, t = 0.255; Fig 3B), suggesting low basal levels of CP-AMPARs in both genotypes. CP-AMPARs are inwardly rectifying at positive voltages resulting in a non-linear current-voltage (IV) relationship. The AMPA IV relationship was therefore measured in the presence of AP-V and BIC, and the peak eEPSC at -70. -50, -30, -10, 10 and 40 mV recorded (Fig 3C). All data were normalized to the peak current at -70 mV. This revealed a linear relationship (WT: n = 6 cells, 2 mice: R^2^ = 0.996, slope = 1.339 ± 0.0402, KO: n = 8 cells, 4 mice: R^2^ = 0.998, slope = 1.292 ± 0.0291) that was unaffected by genotype (WT vs. KO: p = 0.716, F_1,80_ = 0.133), further showing that basal CP-AMPARs were not present in WT or KO MSNs.

#### NMDAR subunit composition

The composition of NMDARs, in particular those containing the GluN2B subunit, can alter glutamatergic synaptic strength [45, 46]. We therefore measured evoked NMDAR currents at +40mV in the presence of CNQX and BIC in the absence, and then presence, of a specific GluN2B inhibitor, Ro 25–6981 [47]. GluN2B-containing NMDAR currents, measured by digital subtraction were lower in KO (n = 8 cells, 3 mice) than WT (n = 7 cells, 4 mice) MSNs (p < 0.05, t = 2.602; Fig 3D).

#### LIMK and βarr1 regulate the AMPA/NMDA ratio and NMDAR subunit composition

**Fig 4:**
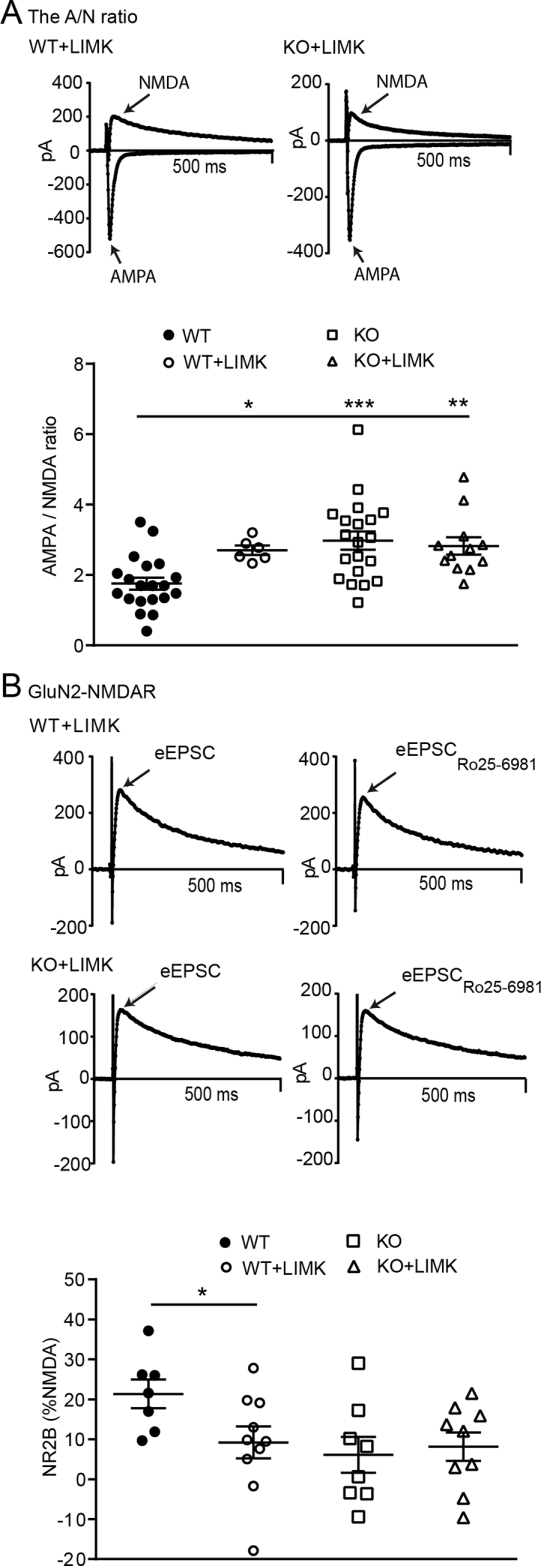
Infusing LIMK in WT MSNs mimics the changes in glutamatergic function seen in KO MSNs. **A)** Inclusion of LIMK in the internal recording solution increased the A/N ratio in WT MSNs (p < 0.05), but did not alter the KO ratio (WT: n = 20, WT-LIMK: n = 6, KO: n = 12, KO-LIMK: n = 12). **B)** Intracellular LIMK also reduced the percent of GluN2B-NMDAR currents in WT MSNs (p < 0.05) without altering that of KO MSNs (WT: n = 10, KO: n = 9). *, **, *** = p < 0.05, 0.01 and 0.001respectively vs WT.

As βarr1 plays an important role in regulating actin turnover to modulate exocytosis and endocytosis [9, 20, 48], it is possible that actin dynamics may be involved in regulating the trafficking of GluN2B-NMDARs. One way to probe this mechanism is to inhibit cofilin activity by adding recombinant LIMK (2μg/ml) to the intracellular recording solution so as to prevent actin severing and subsequent re-organization [11, 49]. Using 2-way ANOVA to assess the effect of LIMK in MSNs from KO and WT mice, we found an interaction of Genotype and LIMK (F_1,54_ = 4.12, p = 0.046). In WT, but not KO MSNs, LIMK increased the A/N ratio (p<0.05, WT: n = 20 cells, 6 mice, WT-LIMK: n = 6 cells, 3 mice KO: n = 20 cells, 5 mice, KO-LIMK: n = 12 cells, 3 mice, Fig 4A). We also examined whether excess LIMK would alter GluN2B-NMDAR currents in a genotype-dependent manner (Fig 4B). Two-way ANOVA showed a trend towards a Genotype x Treatment interaction (F_1,30_ = 3.141, p = 0.0865) and a main effect of Genotype (F_1,30_ = 4.143, p = 0.0507). Post-hoc analyses showed that LIMK reduced the GluN2B component of NMDAR currents in WT MSNs (WT: n = 7 cells, 4 mice vs. WT LIMK: n = 10 cells, 3 mice: p < 0.05). However, LIMK did not alter GluN2B-NMDAR currents in KO MSNs (KO: n = 8 cells, 3 mice vs. KO-LIMK: n = 9 cells, 3 mice). These data suggest that βarr1 and LIMK may regulate both the A/N ratio and GluN2B-NMDARs in WT NAc MSNs.

#### Cocaine self-administration increases the A/N ratio

**Fig 5:**
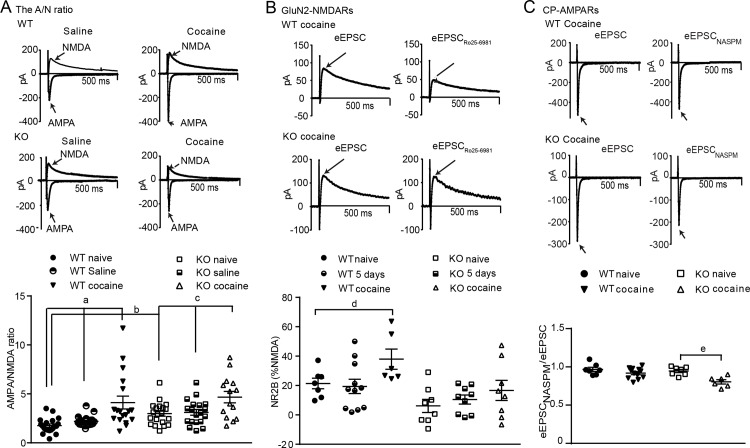
Cocaine self-administration followed by a limited extinction protocol reveals specific deficits in glutamatergic signaling in KO NAc MSNs. **A)** The A/N ratio increased in both WT and KO MSNs following cocaine (WT: n = 18, KO: n = 14), but not after saline (WT: n = 20, KO: n = 19), self-administration. Although KO MSNs showed a higher initial A/N ratio, the post-cocaine ratio was similar in both groups. **B)** WT MSNs showed a cocaine-induced increase in GluN2B-containing NMDARs after the full self-administration protocol (WT: n = 6, KO n = 8; p < 0.05), but not during the early phase (5 days) of acquisition alone (WT: n = 11, KO: n = 9). In contrast, KO MSNs showed lower basal GluN2B-NMDAR currents, as compared to WT MSNs (p < 0.05), which were not altered following cocaine self-administration + extinction. **C)** MSNs from the cocaine self-administering (cocaine self-administration + extinction; WT: n = 12, KO: n = 6) KO mice showed an increase in CP-AMPARs when compared to MSNs from naive WT or KO mice (p < 0.05). CP-AMPAR currents were not detected in WT MSNs from naïve or cocaine groups. a = p < 0.001; b = p < 0.0001; c = p < 0.01; d = p < 0.05 vs WT naïve, e = p<0.05 vs KO naive.

The elevated A/N ratio in naïve KO MSNs could alter the known effect of cocaine in increasing this ratio [26, 50]. We therefore assessed the A/N ratio of NAc shell MSNs of mice that underwent either saline or cocaine self-administration followed by 5 days of extinction (Fig 5A). Using 2-way ANOVA for analysis, we found an effect of Time (F_2,105_ = 15.52, p<0.001) and Genotype (F_2,105_ = 8.24, p = 0.005). In WTs, the A/N ratio was not altered following saline self-administration, (n = 20 cells each for naïve; 6 mice, and saline; 4 mice) but was increased above naïve (p<0.001) or saline (p<0.001) levels following cocaine self-administration + extinction (n = 18 cells, 7 mice). Similar to WTs, the A/N ratio from KOs showed no effect of saline (n = 19 cells, 6 mice) compared with the naïve group (n = 20 cells, 5 mice), and cocaine self-administration + extinction (n = 14 cells, 5 mice) increased this ratio to WT levels when compared with naïve (p<0.05) or saline (p<0.05) groups.

#### Cocaine self-administration results in a greater contribution of GluN2B-containing NMDARs in WT than KO MSNs

Given the relative lack of GluN2B-NMDARs in naïve KO MSNs and the known effect of cocaine in increasing GluN2B-enriched NMDARs [29, 51],we proposed that KO MSNs may not show this change. The peak evoked NMDAR current was measured in the presence of BIC and CNQX, without, and then with the GluN2B inhibitor, Ro25-6981 at 2 time points; after 5 days of cocaine self-administration, and at the end of the acquisition and the 5-day extinction period (Fig 5B). Using 2-way ANOVA for analysis, we found a Time (F _2,43_ = 3.92, p = 0.03) and Genotype effect (F _2,43_ = 12.93, p = 0.0008). For the WTs, no change in GluN2B-containing NMDAR currents was seen after 5 days but there was an effect of the full cocaine protocol (p<0.05, n = 7 cells (4 mice), 11 cells (3 mice), 6 cells (3 mice) for naïve, 5-day and cocaine self-administration +extinction groups respectively). In contrast, KOs showed no effect of cocaine after either 5-days or the full self-administration protocol (n = 8 cells (3 mice), 9 cells (3 mice), 8 cells (3 mice) for naïve, 5-day and cocaine self-administration + extinction groups respectively).

#### Cocaine self-administration increases the contribution of CP-AMPARs in KO but not WT NAc neurons

Cocaine self-administration followed by extinction can result in the insertion of post-synaptic CP-AMPARs [52]. We therefore examined whether CP-AMPARs were present in KO or WT MSNs following cocaine self-administration + extinction (Fig 5C). The peak AMPAR current (measured at -70mV, in the presence of BIC and AP-V) was assessed in the absence, and then presence, of NASPM. Using 2-way ANOVA for analysis, we found an effect of the intervention, cocaine self-administration + extinction, in KO, but not WT (F_1,28_ = 6.73, p = 0.015) MSNs. The KOs showed a decrease in peak AMPAR current in the presence of NASPM after the full cocaine self-administration protocol, suggesting an increased presence of CP-AMPARs (naïve; n = 8 cells, 4 mice, cocaine; n = 6 cells, 3 mice: p < 0.05). In contrast, the same cocaine self-administration protocol did not alter the response to NASPM in WT MSNs (naïve; n = 7 cells, 3 mice, cocaine; n = 12 cells, 4 mice), possibly due to the short extinction period [53]. In summary, cocaine self-administration followed by a short period of extinction increased the relative proportion of AMPAR to NMDAR currents in both WT and KO mice, but this was achieved by a different glutamatergic subunit composition in KO than WT MSNs.

### Inhibiting GluN2B-containing NMDARs in the NAc alters food-reinforced instrumental conditioning

**Fig 6:**
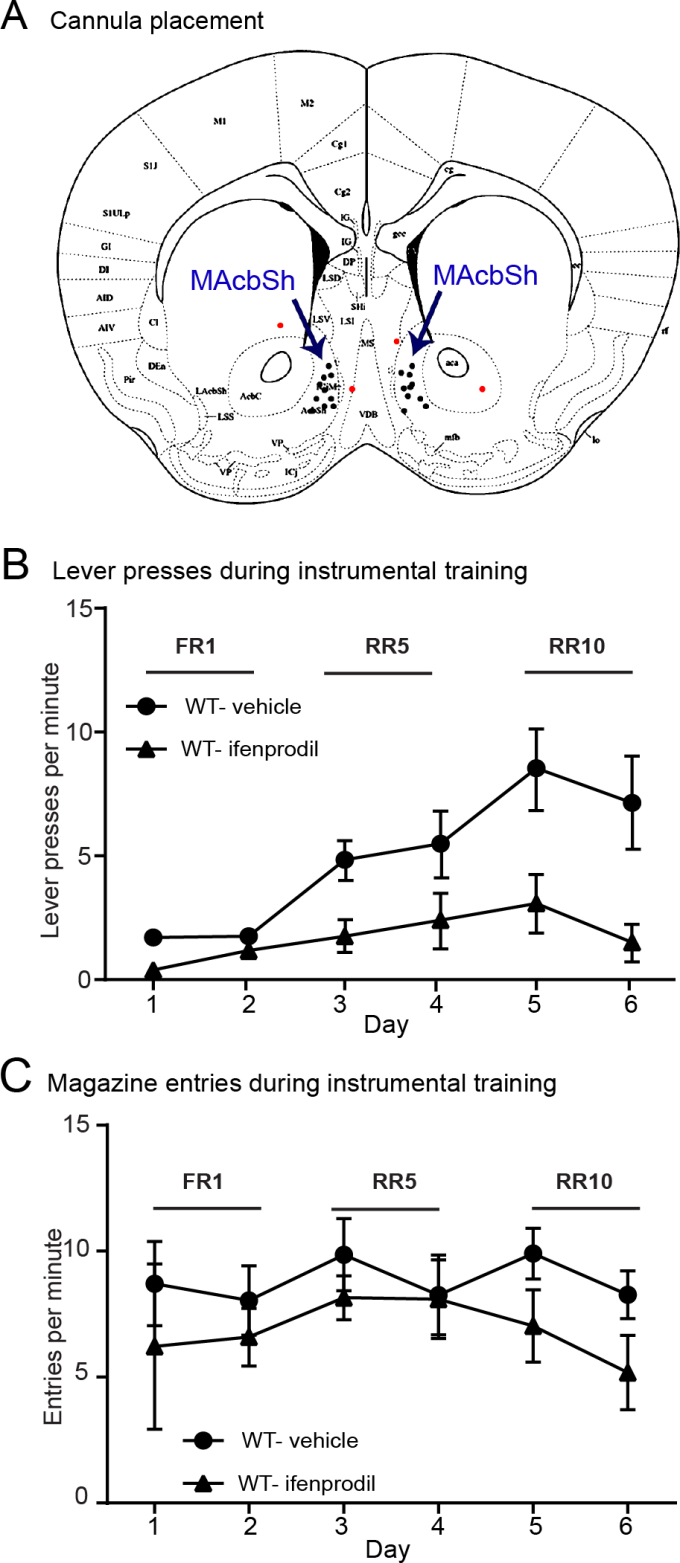
Chronic inhibition of GluN2B-NMDARs disrupts food-reinforced instrumental conditioning in WT mice. **A)** A schematic depiction showing the placement of bilateral cannulae in the medial NAc shell (MAcbSh) for ifenprodil infusion. Two mice, shown as red and orange pairs of dots, were removed from the experiment due to misplaced cannulae. **B)** Ifenprodil-treated mice had a lower rate of level pressing than vehicle treated mice (main effect of treatment: p < 0.05). **C)** Ifenprodil-treatment did not alter the rate of magazine entries during these instrumental conditioning sessions (Vehicle: n = 7, Ifenprodil: n = 4).

As KO mice show an impairment in performing an effortful food-seeking task and a reduction in GluN2B-NMDARs, we next explored this relationship by inhibiting this NMDAR subunit in the NAc shell of WT mice undergoing tests of food reward. Vehicle or ifenprodil, a GluN2B subunit selective antagonist, was chronically infused through bilateral intra-accumbal cannulae connected to a subcutaneous osmotic pump. For these experiments, we focused on food, rather than cocaine, motivated behaviors as it is not possible to implant both an intravenous catheter with its stabilizing back-mount and a subcutaneous osmotic pump connected to bilateral intra-cranial cannulae in mice. We also used a shortened version of the initial food reward behaviors due to the 14-day limit of drug delivery by the osmotic pump. After initial pre-training to consume 20% sucrose solution from the magazine, mice were given 6 days of instrumental conditioning to lever press for sucrose with an incremental reinforcement schedule; 2 days at FR1, 2 days at RR5 and 2 days at RR10. Cannula placement was then verified and 2 mice receiving ifenprodil were removed from the dataset as the cannula were outwith the NAc shell (ifenprodil; n = 7, vs. vehicle, n = 4]; Fig 6A). A mixed ANOVA detected a significant main effect of Treatment (F_1,9_ = 6.737; p = 0.029), main effect of Day (F_5,45_ = 7.683; p < 0.001), and Treatment x Day interaction (F_5,45_ = 2.547; p = 0.041), indicating ifenprodil suppressed instrumental lever pressing (see Fig 6B). In this experiment, ifenprodil-treated mice were capable of acquiring a level of performance similar to vehicle-treated mice when response demands were low (FR1), but exhibited a much lower asymptotic rate of pressing when the effort needed to earn reward was increased (RR5-10; Fig 6B). This suppression of the ifenprodil-induced response appeared specific to instrumental lever-pressing behavior. A separate ANOVA on the rate of magazine entry behavior during these instrumental conditioning sessions (Fig 6C) found no effect of ifenprodil (F_1,9_ = 1.296, p = 0.284) and no Treatment x Day interaction (F_5,45_ = 0.461; p = 0.803), indicating that the behavioral suppression induced by ifenprodil was not due to a generalized motor deficit. In summary GluNB inhibition in the NAc shell inhibited instrumental lever pressing behavior in WT mice.

## Discussion

In this study we examined the role of βarr1, a ubiquitously expressed scaffolding and signal transduction molecule, in reward-related behaviors and glutamatergic function. Using mice lacking βarr1, we found a delay in the initial phases of both the acquisition and extinction of cocaine self-administration in the absence of this protein. These KO mice also showed an impairment in developing Pavlovian conditioned (goal) approach behavior and in the amount of effort needed to obtain food reward. As βarr1 may be involved in the trafficking to, and stability of glutamatergic receptors on the cell membrane and the glutamatergic system is an integral component of reward behaviors [54], we then examined the effect of deleting βarr1 on glutamatergic signaling in the NAc shell. We found a basal increase in the A/N ratio and a relative loss of functional GluN2B-NMDARs in MSNs of this region. Cocaine self-administration induced an increase in synaptic strength in both KO and WT MSNs but this was achieved by a different combination of ionotropic receptor subunits in KO vs WT neurons. In order to assess the role of reduced GluN2B-NMDARs in reward behaviors, we chronically inhibited these subunits in the NAc shell of WT mice during an instrumental food training paradigm. This resulted in an attenuation of food-motivated behaviors in WT mice. Together these data outline a role for βarr1 in regulating reward-motivated behaviors and glutamatergic function.

βarr1 KOs showed deficits in the initial phases of food and cocaine behaviors, however they reached the same asymptote as WTs after a few days. KOs exhibited a persistent attenuation of conditioned goal-approach behavior during food-reinforced Pavlovian conditioning, but it is important to note that this aspect of behavior does not provide a pure measure of Pavlovian learning since animals must perform this task during training in order to obtain food reward [55]. Importantly, no deficits were observed in food-motivated PIT performance measures which provide more conclusive tests of Pavlovian (stimulus-reward) learning as they focus on how reward-paired cues influence new behaviors. When considered together, the relatively normal performance that βarr1 KO mice exhibited on these tasks suggests that although βarr1 may not be required for learning, *per se*, or processing the affective/motivational value of rewards, this scaffolding molecule plays a more fundamental role in the regulation of effortful reward-seeking behavior. The lack of an effect of deleting βarr1 at more demanding schedules could reflect a differential sensitivity to cocaine that may mask any differences in the motivation to obtain this drug at more demanding schedules (FR2 and FR5). A complete dose response curve would be needed to examine this possibility.

We found that deleting βarr1 increased the A/N ratio and reduced GluN2B-NMDARs in NAc shell MSNs. These observations could be mimicked by manipulating LIMK in WT, but not KO MSNs, suggesting that both LIMK and βarr1 are involved in regulating glutamatergic synaptic strength and NMDAR subunit composition in WT mice. βarr1, as a scaffold for LIMK, SSL or CIN and cofilin regulates actin turnover and receptor export [9, 11, 19]. The actin cytoskeleton is also involved in regulating AMPAR and NMDAR localization and function [18, 56, 57]. Based on our findings it is possible that deleting βarr1, or inactivating cofilin by providing excess LIMK, disrupts actin turnover and microtubule disassembly to reduce the transport of GluN2B-containing NMDARs to the cell membrane, or stability once in place. Our data show that LIMK and βarr1 regulate GluN2B-NMDARs but it also known that NMDARs, and in particular GluN2B-NMDARs, may in turn regulate actin turnover and treadmilling to influence dendritic spine remodeling [58] in an arrestin-dependent manner [59]. This outlines a complex interaction whereby GluN2B-NMDARs may require the actin cytoskeleton for correct membrane anchorage and function but, once activated, NMDARs also regulate the actin cytoskeleton. However, further studies are needed to fully explore this relationship between ionotropic receptor trafficking, the actin cytoskeleton and βarr1.

The short-access cocaine self-administration followed by 5 days of extinction increased the A/N ratio without altering the presence of CP-AMPARs in WT mice. This is in accord with the findings of others where short access to cocaine and relatively short extinction protocols do not increase GluA1-containing AMPARs [28, 53]. In contrast, our cocaine self-administration protocol did increase CP-AMPARs in KO MSNs. This could have been a direct result of GluN2B-induced regulation of GluA1 subunits whereby the relative paucity of GluN2B-NMDARs facilitates the appearance of CP-AMPARs [60]. It is also possible that the increase in CP-AMPARs following cocaine self-administration was a direct result of deleting βarr1. In regulating F-actin disassembly and subsequent G-actin reorganization, cofilin, a βarr1 binding partner, is intimately involved in CP-AMPAR trafficking [14, 49, 61]. Deleting βarr1 could feasibly alter CP-AMPAR trafficking by this mechanism.

Given the evidence that the deletion of βarr1 disrupts GluN2B-NMDAR function, we examined whether chronic inhibition of GluN2B-NMDARs in the NAc shell would produce a similar behavioral impairment as seen in βarr1 KOs. We found that continuous infusion of a selective GluN2B-NMDARs antagonist into the NAc shell of WT mice led to a disruption of food-reinforced lever pressing behavior that was similar, but also differed from, the deficit seen in βarr1 KOs. These differences could reflect 1) the relative levels of these receptors in each intervention; whereas chronic ifenprodil infusion likely inhibited all GluN2B-NMDARs, Figs 4B and 5B show some level of these receptors in KO neurons, and, 2) the different experimental protocol used due to the 14 day limit of osmotic pump drug delivery. Although we did not include KO mice in these technically demanding experiments, the fact that chronic inhibition of these subunits in WT mice induced an attenuation of lever pressing behavior, as was seen in the KO mice, albeit to a lesser extent, implies a relationship between GluN2B dysregulation and impaired reward processing. However, the causal nature of this relationship will need to be explored in future work.

### Conclusion

We have found that deleting βarr1 results in specific deficits in cocaine and food-related behaviors and in glutamatergic synaptic strength and subunit composition. Our initial assessment is that these deficits are related to a βarr1-dependent trafficking of GluN2B-NMDARs along the actin cytoskeleton. Although further studies are needed to fully define the underlying cellular mechanisms and causal relationships, this study is the first to examine the role of this arrestin isoform in reward-motivated behaviors.

## Supporting information

S1 Table The number of mice removed from each stage of the cocaine IVSA experiment. (DOCX)

S1 Fig Survival plot of mice reaching acquisition criteria at FR1 of cocaine IVSA. WT mice (n = 22) reached acquisition criteria faster than the KO mice (n = 19) during the FR1 stage of cocaine self-administration experiments (p < 0.05).
(TIF)

S2 Fig Individual datapoints for the cocaine IVSA experiment shown in Fig 1. The number of cocaine infusions earned by KO and WT mice during the **A)** first 5 days of FR1 and during **B)** the last 3 days of FR1, FR2 and FR5. The number of within-session active lever presses during **C**) the fifth day of FR1, **D)** the first day of FR2 and **E)** the first day of FR5 in 30-minute bins. **F)** Total lever pressing during the extinction stage. **G)** Total lever presses during the first day of extinction in 30-minute bins.
(TIF)

S3 Fig Individual datapoints for the food-reinforced Pavlovian and instrumental training experiment shown in Fig 2. **A)** Magazine entries per minute during Pavlovian training. **B)** Lever presses per minute during instrumental training sessions. **C**) Daily lever presses per minute averaged across the three random ratio schedules. **D)** Lever pressing rate during the PIT test. **E)** Magazine entry rate during the PIT test.
(TIF)

S4 Fig Individual datapoints for the IV curve in WT and KO neurons. (TIF)

S5 Fig Individual datapoints for the chronic inhibition of GluN2B-NMDAR experiment shown in Fig 6. **A)** Lever presses per minute during instrumental training. **B)** Magazine entry rate during instrumental training sessions.
(TIF)
