# Supplementary material for: Beta-arrestin 1 regulation of reward-motivated behaviors and glutamatergic function
Source: PLoS One. 2017 Oct 3;12(10):e0185796. doi: 10.1371/journal.pone.0185796 (PMC5626489; doi:10.1371/journal.pone.0185796)
Supplement: S1 Table — (DOCX) [file pone.0185796.s001.docx]

**S1 Table. The number of mice removed from each stage of the cocaine IVSA experiment**. The number of animals that lost patency ^(a)^, failed to reach the acquisition scriteria^(b)^, or were removed for electrophysiology studies^(c)^, are reported below. The numbers in parentheses represent the number of subjects at the beginning of each of these experimental stages.

|  | FR1 before day 5 | FR1 after day 5 | FR2 | FR5 | Extinction |
| --- | --- | --- | --- | --- | --- |
| WT mice (26) | 4^a^ (22) | 0^a^ (22) | 6^a^ 2^b^ (14) | 1^a^ (13) | 9^c^ (4) |
| KO mice (23) | 2^a^ (21) | 2^a^ (19) | 5^a^ 2^b^ (13) | 2^a^ (11) | 5^c^ (6) |
