# Supplementary figures and images for: Beta-arrestin 1 regulation of reward-motivated behaviors and glutamatergic function
Source: PLoS One. 2017 Oct 3;12(10):e0185796. doi: 10.1371/journal.pone.0185796 (PMC5626489; doi:10.1371/journal.pone.0185796)

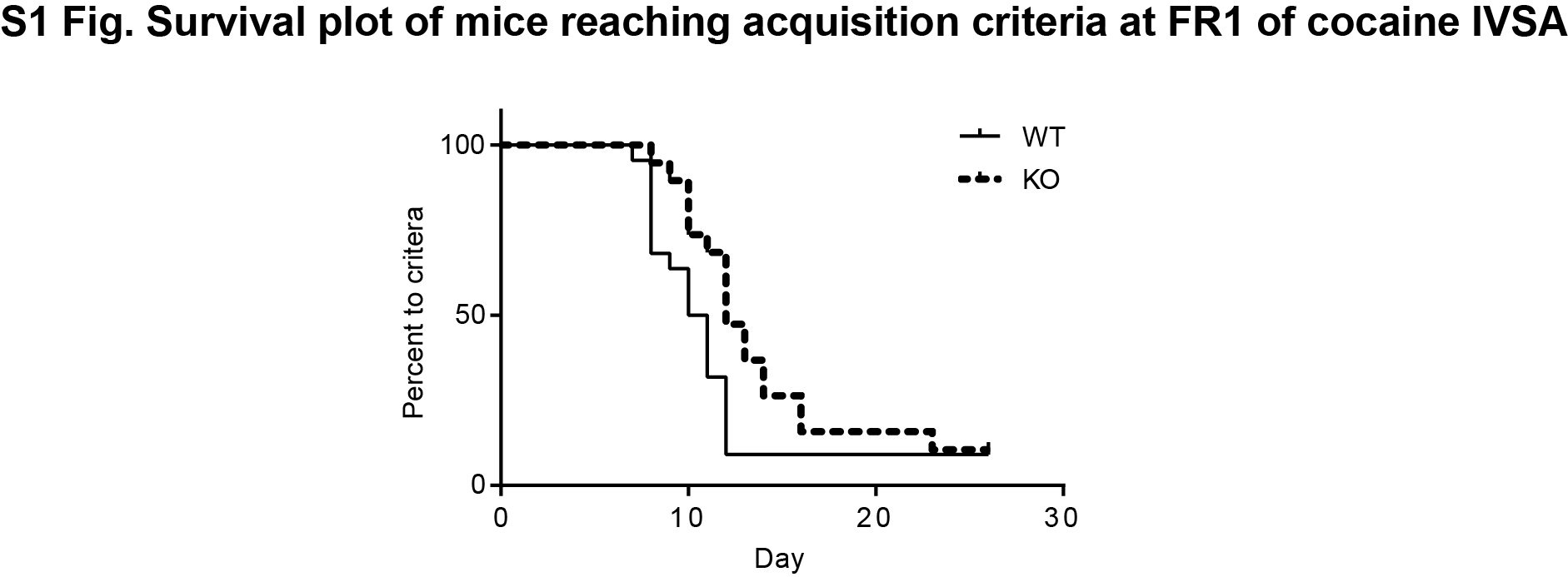

Supplement: S1 Fig — WT mice (n = 22) reached acquisition criteria faster than the KO mice (n = 19) during the FR1 stage of cocaine self-administration experiments (p < 0.05). (TIF) [file pone.0185796.s002.tif]

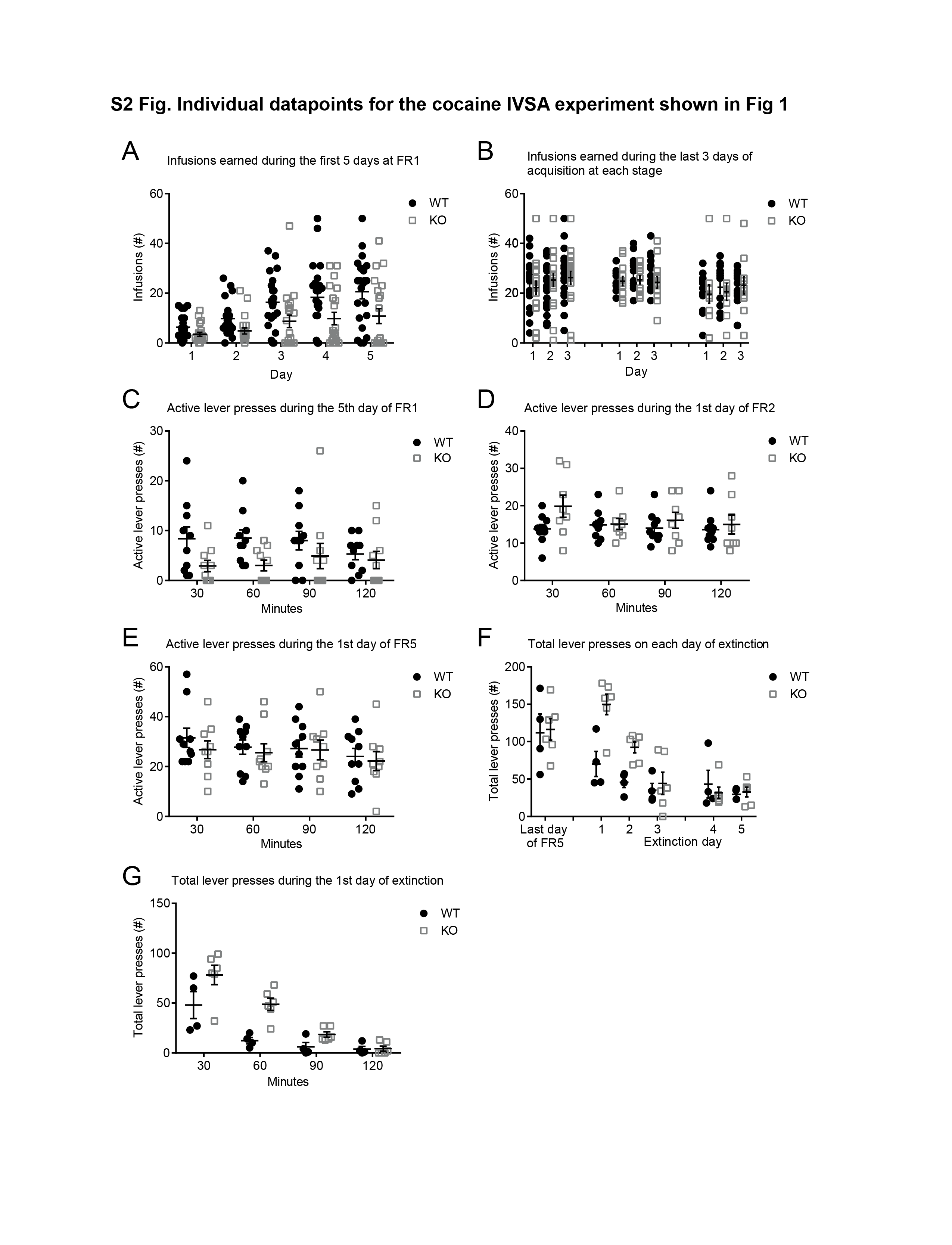

Supplement: S2 Fig — The number of cocaine infusions earned by KO and WT mice during the A) first 5 days of FR1 and during B) the last 3 days of FR1, FR2 and FR5. The number of within-session active lever presses during C) the fifth day of FR1, D) the first day of FR2 and E) the first day of FR5 in 30-minute bins. F) Total lever pressing during the extinction stage. G) Total lever presses during the first day of extinction in 30-minute bins. (TIF) [file pone.0185796.s003.tif]

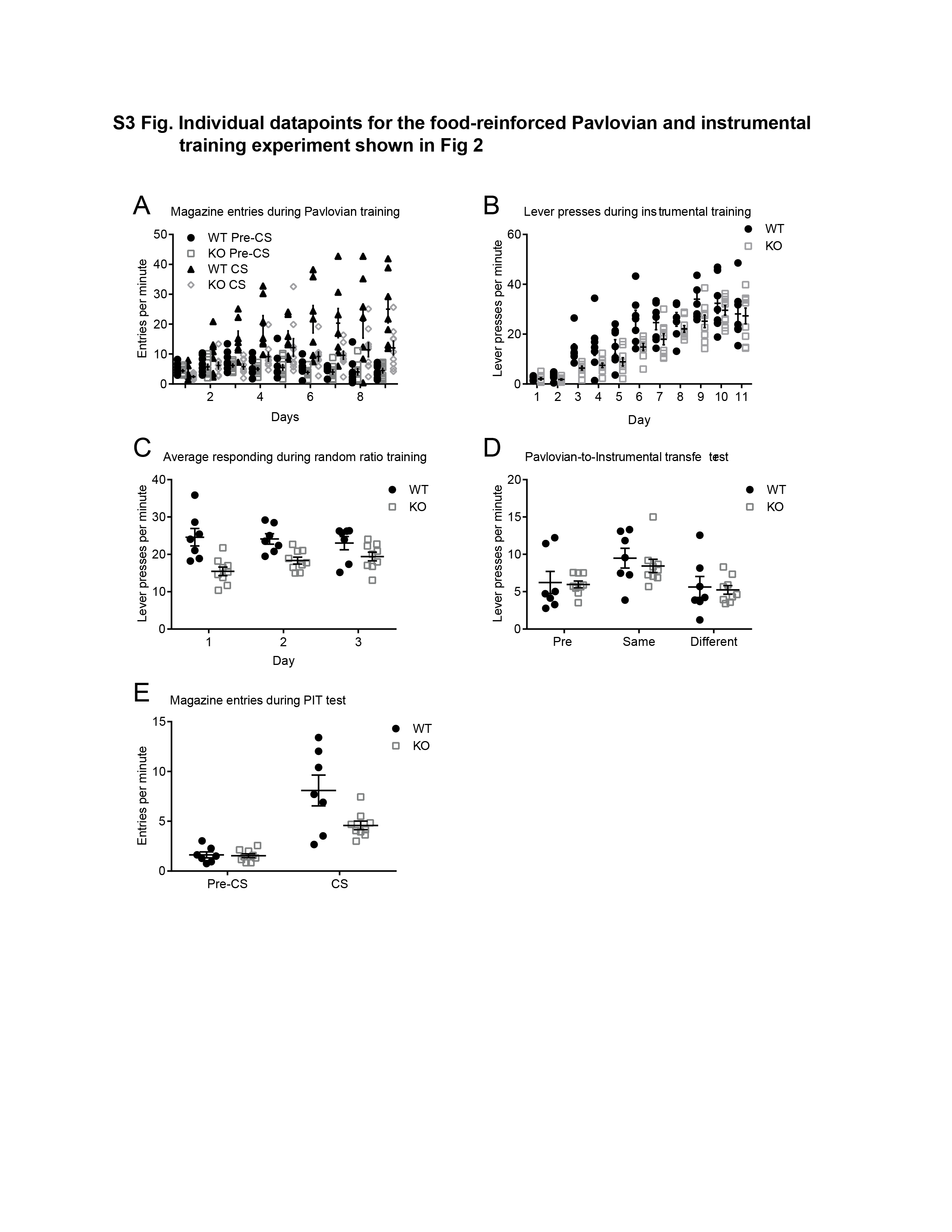

Supplement: S3 Fig — A) Magazine entries per minute during Pavlovian training. B) Lever presses per minute during instrumental training sessions. C) Daily lever presses per minute averaged across the three random ratio schedules. D) Lever pressing rate during the PIT test. E) Magazine entry rate during the PIT test. (TIF) [file pone.0185796.s004.tif]

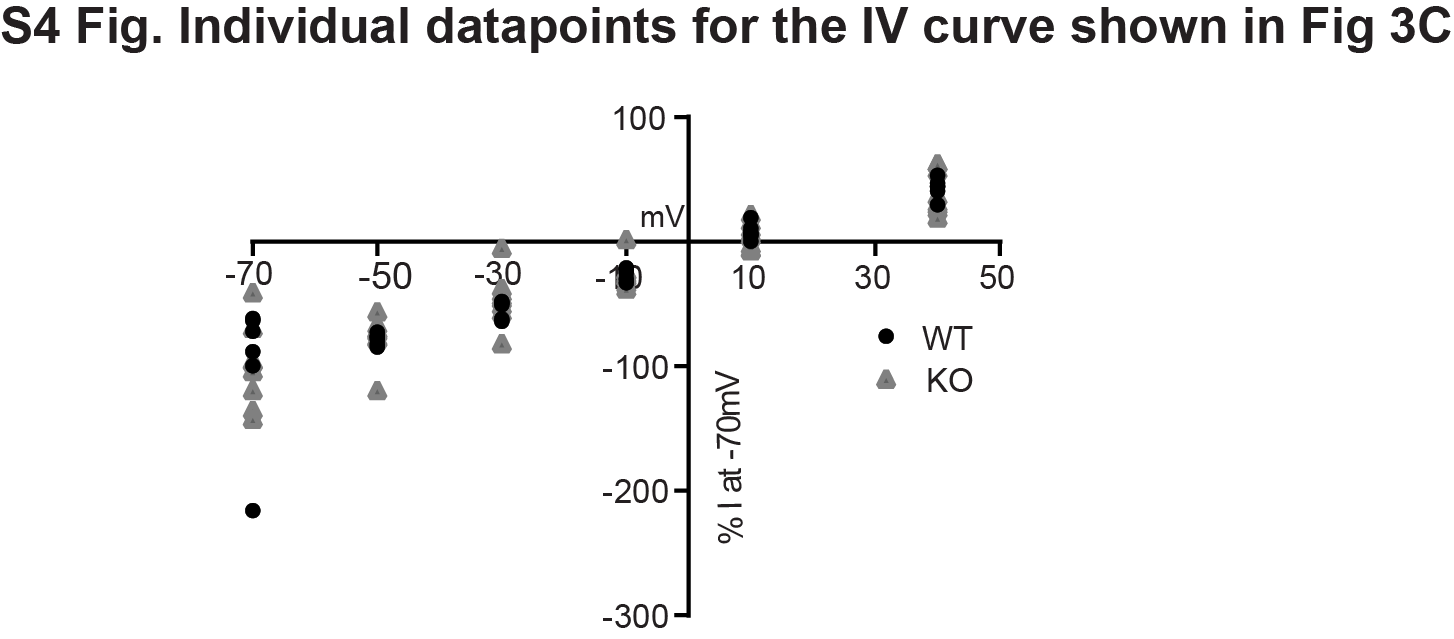

Supplement: S4 Fig — (TIF) [file pone.0185796.s005.tif]

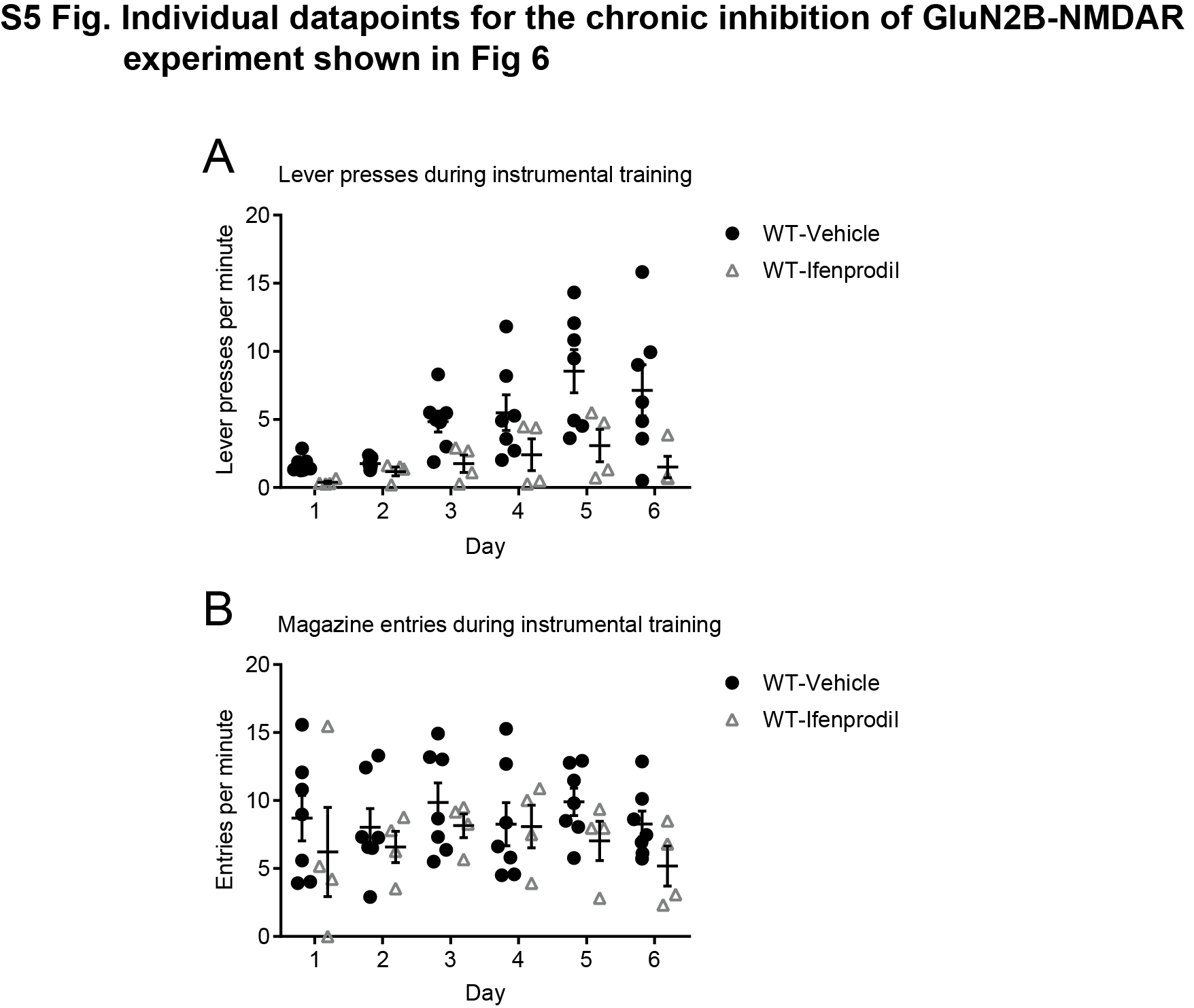

Supplement: S5 Fig — A) Lever presses per minute during instrumental training. B) Magazine entry rate during instrumental training sessions. (TIF) [file pone.0185796.s006.tif]
